# Association between blood pressure variability and clinical outcomes after successful recanalization in patients with large vessel occlusion stroke after mechanical thrombectomy

**DOI:** 10.3389/fneur.2022.967395

**Published:** 2022-08-10

**Authors:** You Lu, Rui Shen, Wenjian Lin, Xiaoyu Zhou, Jian Hu, Quanbin Zhang

**Affiliations:** ^1^Department of Neurology, Shanghai Tenth People's Hospital, Tongji University School of Medicine, Shanghai, China; ^2^Department of Neurosurgery, Shanghai Tenth People's Hospital, Tongji University School of Medicine, Shanghai, China; ^3^Department of Radiology, Shanghai Tenth People's Hospital, Tongji University School of Medicine, Shanghai, China

**Keywords:** blood pressure variability, recanalization, large vessel occlusion, stroke, mechanical thrombectomy

## Abstract

**Objective:**

Nearly half of patients who undergo mechanical thrombectomy (MT) do not experience a favorable outcome. The association between blood pressure fluctuation and clinical outcomes after successful MT is controversial. We evaluated the influence of blood pressure variability (BPV) on the clinical outcomes of stroke patients with large vessel occlusion (LVO) who underwent successful recanalization after MT.

**Methods:**

Patients with anterior circulation LVO stroke who underwent successful emergency MT (modified Thrombolysis in Cerebral Infarction, mTICI ≥ 2b) at the Shanghai Tenth People's Hospital of Tongji University from 2017 to 2021 were enrolled. Multivariate logistic models were used to investigate the association between BPV (mean arterial pressure [MAP] assessed using the standard deviation [SD]) and clinical outcomes. The primary outcome was 90-day modified Rankin Scale scores (mRS), and the secondary outcomes were 30-day mortality and symptomatic intracranial hemorrhage (sICH).

**Results:**

A total of 458 patients (56.8% men), with a mean age of 72 ± 1 years, were enrolled. Among them, 207 (45.2%) patients had unfavorable functional outcomes (mRS score 3–6) at 90 days, 61 (13.3%) patients died within 30 days, and 20 (4.4%) patients had sICH. In a fully adjusted model, BPV was associated with a higher risk of a 90-day mRS score of 3–6 (*P* = 0.04), 30-day mortality (*P* < 0.01), and sICH (*P* < 0.01). A significant interaction between MAP SD and rescue futile recanalization treatment was observed (*P* < 0.01).

**Conclusions:**

Among patients with LVO stroke who underwent successful recanalization, higher BPV was associated with worse functional outcomes, especially in those who underwent rescue treatment.

## Introduction

Large vessel occlusion (LVO) stroke is associated with a 4.5-fold increased risk of mortality compared to non-LVO stroke (1). Mechanical thrombectomy (MT) is the most effective treatment method for patients with LVO stroke and has a one-fold higher recanalization rate when compared to intravenous thrombolysis for patients with acute ischemic stroke caused by anterior circulation LVO. However, nearly half of patients with successful recanalization fail to achieve functional improvement (2).

Hypertension is one of the most common risk factors for stroke (3). Most observational studies have suggested that increased blood pressure (BP) after MT increases mortality and symptomatic intracranial hemorrhage (sICH) (4, 5). However, BP management after endovascular therapy remains a clinical challenge. The American Stroke Association (ASA) guidelines recommend maintaining a BP level of <180/105 mmHg for 24 h after MT, based on low-level evidence (6). In the DAWN trial, a systolic blood pressure (SBP) of <140 mmHg is maintained in the first 24 h in patients with successful reperfusion (defined as achieving a modified Thrombolysis in Cerebral Infarction (mTICI) grading system score of 2b-3) after MT (7). Contrarily, 62% of the stroke centers in the USA set a target SBP of <160 mmHg for patients with successful reperfusion (8). There were no significant differences in the efficacies between the intensive and non-intensive arms in the BP-TARGET trial, suggesting BP parameters other than SBP may be closely associated with clinical outcomes (9).

Blood pressure variability (BPV) has been reported to be a better predictor of all-cause and cardiovascular mortality and cardiac disease than other BP paraments (10). The influence of BPV on clinical outcomes in stroke patients has recently attracted growing attention (11, 12). However, conflicting conclusions have been reported regarding the association between BPV and clinical outcomes after MT. A secondary analysis of the prospective cohort study BEST revealed that higher BPV is associated with exacerbated 90-day outcomes (13), and yet post hoc analyses of the random clinical trial BP-TARGET suggested that BPV was not associated with functional outcomes (14).

Therefore, we performed a retrospective cohort study to investigate the association between BPV after successful recanalization and clinical outcomes in patients with LVO after MT.

## Methods

### Study population

This study was a retrospective cohort analysis conducted at a comprehensive stroke center (Shanghai Tenth People's Hospital, Tongji University). Consecutive patients with anterior circulation LVO stroke who underwent emergency MT between January 2017 and December 2021 were enrolled. The study was approved by the local ethics committee (No. SHSY-IEC-4.1/21-208/01).

**Figure 1:**
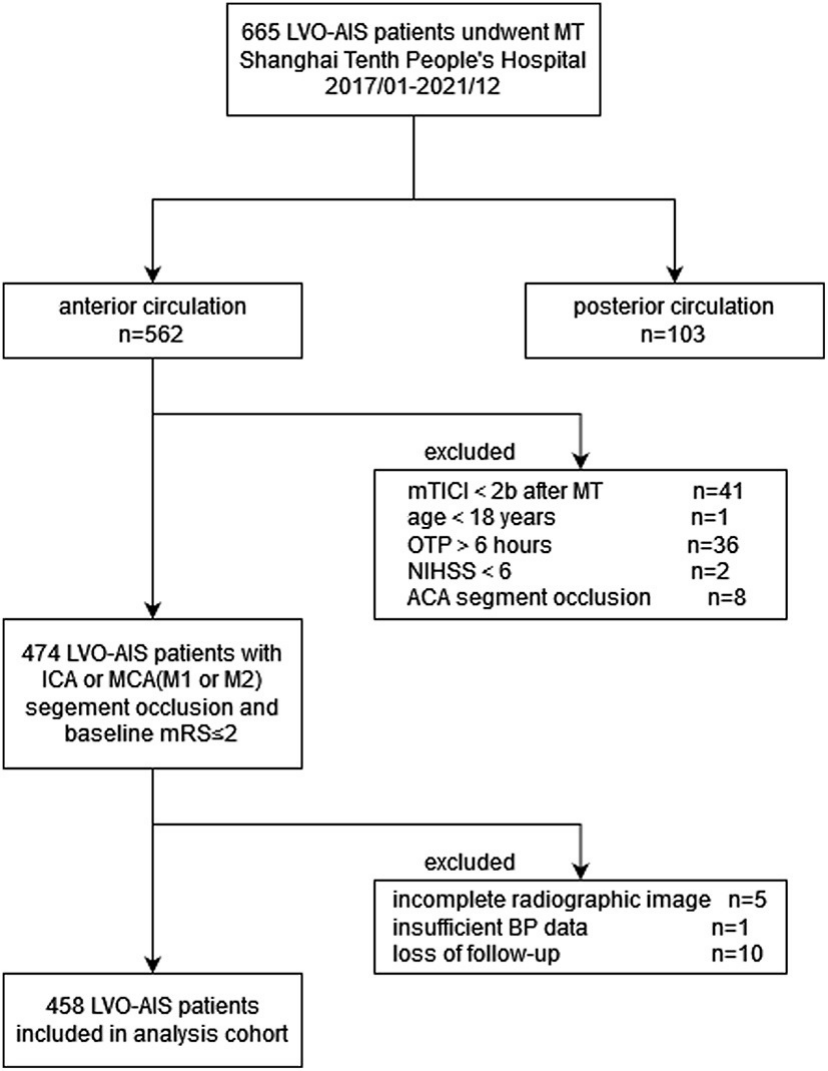
Flow chart of patient inclusion. LVO, Large vessel occlusion; AIS, acute ischemic stroke; MT, mechanical thrombectomy; mTICI, modified tissue thrombolysis in cerebral ischemia; OTP, onset to Puncture time (minute); NIHSS, national institutes of health stroke scale; ACA, anterior cerebral artery; ICA, internal carotid artery; MCA, middle cerebral artery; mRS, modified Rankin Scale; BP, blood pressure.

Patients were registered if they met the following inclusion criteria: (1) aged ≥18 years; (2) time from stroke onset to puncture (OTP) was ≤6 h; (3) baseline National Institutes of Health Stroke Scale (NIHSS) score of ≥6, baseline Alberta Stroke Program Early computed tomography (ASPECT) score of ≥6 and pre-stroke modified Rankin Scale (mRS) score of <2; (4) occlusion of the internal carotid artery, proximal segment (M1/M2) of the middle cerebral artery confirmed by computed tomography angiography or digital subtraction angiography imaging; (5) successful recanalization, defined as an mTICI score of 2b or 3 after MT. Patients were not enrolled based on the following exclusion criteria: (1) insufficient BP data (recorded for <24 hours); (2) incomplete radiographic images; and (3) absence of follow-ups. The flow chart of the study population inclusion is shown in Figure 1.

### Study definitions

LVO in the anterior circulation was defined as occlusion of the internal carotid artery or middle cerebral artery segment M1 or proximal M2 (2). Rescue treatment was defined as balloon angioplasty and/or stenting (15). The recanalization status of the patients was evaluated based on the mTICI grading system. Successful recanalization was defined as an modified Thrombolysis in Cerebral Infarction (mTICI) score of 2b or 3 (16, 17).

Although the use of continuous intravenous antihypertensive agents had not been previously defined in the data, steady-state use was defined as long-term use. In the Chinese guidelines for endovascular treatment, recommendations for BP management include maintaining a BP of <140/90 mmHg while avoiding one of <100/60 mmHg.

### Data collection

According to previous studies (13, 14, 18, 19), demographic characteristics (sex and age), medical history (hypertension, diabetes, and atrial fibrillation), admission variables (baseline NIHSS score, ASPECT score, and level of occlusion), procedural variables (bridging therapy, puncture to recanalization [PTR] time, onset to recanalization [OTR] time, rescue treatment, and postoperative continuous intravenous therapy), and BP data 24 h after MT were collected.

Demographic characteristics, medical history, baseline NIHSS scores, and BP data (SBP/diastolic blood pressure, [DBP] were obtained from an electronic medical database. If the NIHSS score was not documented in the database, it was extrapolated from documented neurologic examinations using a validated method. The ASPECT score was calculated for these patients by a neurologist and radiologist who were blinded to the patient outcomes.

The procedural parameters were recorded by the operators, including PTR, OTR, occlusion site, bridging, rescue treatment, and continuous intravenous antihypertensive agents administered.

BP was measured at 1-h intervals from admission for the first 24 h after MT. If the patient was out for examination at the time of BP measurement, BP was measured 10 min after returning to the stroke unit. Nurses in the stroke and intensive care units recorded the blood pressure measurements into the electronic medical record. BP data were obtained from an electronic medical database in our study. The standard deviation (SD) of the mean arterial pressure (MAP) was used as the key indexes.


$$\begin{array}{l} \text{MAP }=\;\left(\text{SBP }+\;2\;*\text{ DBP}\right)/3\\ \text{MEAN }=\frac{\text{MA}{\text{P}}_{1}\;+\text{ MA}{\text{P}}_{2}\;+\;\cdots \;+\text{ MA}{\text{P}}_{\text{n}}}{\text{n}}\\ \text{SD }=\sqrt{\frac{1}{\text{n}-1}\overset{\text{n}-1}{\underset{\text{i}=1}{\sum }}{\left(\text{B}{\text{P}}_{\text{i}}\;-\text{ B}{\text{P}}_{\text{mean}}\right)}^{2}} \end{array}$$

### Outcome measures

The primary outcome was futile recanalization, defined as a 90-day mRS score of 3–6 (20).

The secondary outcomes included 30-day mortality and sICH. sICH was defined as any intraparenchymal, subarachnoid, or intraventricular hemorrhage diagnosed by post-endovascular thrombectomy non-contrast head CT with a ≥4 point increase in the NIHSS score within 24 h after endovascular thrombectomy according to the European-Australasian Acute Stroke Study II criteria (21).

Outcome assessments were obtained through clinic or telephone follow-ups if specific documentation was not available in the electronic medical database.

### Statistical analysis

Continuous variables are presented as means ± standard deviation (normal distribution) or median (interquartile range for skewed distribution). Categorical variables are presented as numbers (%). Continuous variables were analyzed using the Kruskal-Wallis test. Categorical variables were analyzed using the Chi-squared test or Fisher's exact test, as appropriate.

Logistic regression models were used to investigate any BPV correlations and to calculate the odds ratios between BPV and each outcome. Associations between BPV and outcomes were assessed using continuous variables with BPV as a continuous variable (per SD). According to the recommendations of the STROBE statement (22), we simultaneously show the results of unadjusted (Crude Model), minimally adjusted (model I, adjusted for demographic characteristics, medical history, and admission variables), and fully adjusted analyses (model II, adjusted for model I and procedural variables). We also used smooth curve fitting (penalized spline method) to identify the nonlinear relationships between the continuous variables (age, baseline NIHSS, PTR time, OTR time, MAP mean) and outcomes. Nonlinear regression (curve fit) was used for the variables that did not fit the linear model. To evaluate the robustness of this study, a subgroup analysis using a logistic model was performed as a sensitivity test. We converted it to a categorical variable according to the clinical cut-off point and then performed an interaction test effect modification for subgroup indicators using the likelihood ratio test.

Data were analyzed using the R statistical packages (The R Foundation; http://www.r-project.org; version 3.4.3) and EmpowerStats (www.empowerstats.com; X&Y Solutions Inc.). Statistical significance was defined as a two-sided *P* < 0.05.

## Results

### General characteristics

From January 2017 to December 2021, 665 consecutive patients were enrolled in the thrombectomy cohort. Among them, 562 (84.5%) were ischemic stroke in the anterior circulation. We enrolled 474 patients in the initial cohort according to the inclusion criteria. In total, 16 patients (3.4%) were excluded from the final analysis because of incomplete radiographic images (*n* = 5), insufficient BP data (*n* = 1, this patient died within 24 h after thrombectomy) and lost to follow-up (*n* = 10). Consequently, 458 patients were analyzed (Figure 1).

The baseline characteristics of all participants according to MAP SD quartiles are shown in Table 1. The mean age of the participants was 71.7 ± 10.7 years, and 56.8% were men. Among them, 74.2% had hypertension, 28.2% had diabetes mellitus, and 46.7% had atrial fibrillation. The mean baseline NIHSS score was 16.0 ± 4.1, PTR time was 47.0 min (30.0–70.0), and onset-to-reperfusion time was 270.1 ± 83.5 min.

**Table 1:** Characteristics of the study population (*n* = 458).

	**MAP SD, mmHg**					
	**ALL**	**Q1: 2.8-7.1**	**Q2: 7.1-8.6**	**Q3: 8.6-10.5**	**Q4: 10.5-26.2**	***P* Value**
	***n* = 458**	***n =* 115**	***n =* 114**	***n =* 114**	***n =* 115**	
**Demographic Characteristics**						
Age, year	71.7 ± 10.7	69.1 ± 10.9	70.6 ± 11.0	72.6 ± 9.9	74.3 ± 10.4	0.001
Sex (male)	198 (43.2%)	40 (34.8%)	45 (39.5%)	53 (46.5%)	60 (52.2%)	0.041
**Vascular risk factors**						
Hypertension	340 (74.2%)	77 (67.0%)	74 (64.9%)	94 (82.5%)	95 (82.6%)	<0.001
Diabetes mellitus	129 (28.2%)	24 (20.9%)	28 (24.6%)	39 (34.2%)	38 (33.0%)	0.067
Atrial fibrillation	214 (46.7%)	51 (44.3%)	57 (50.0%)	51 (44.7%)	55 (47.8%)	0.804
**Admission variables**						
Level of occlusion on DSA imaging						0.739
ICA	185 (40.4%)	43 (37.4%)	43 (37.7%)	46 (40.4%)	53 (46.1%)	
MCA 1	219 (47.8%)	57 (49.6%)	60 (52.6%)	54 (47.4%)	48 (41.7%)	
MCA 2	54 (11.8%)	15 (13.0%)	11 (9.6%)	14 (12.3%)	14 (12.2%)	
ASPECTS						0.024
6–8	261 (57.0%)	53 (46.1%)	63 (55.3%)	72 (63.2%)	73 (63.5%)	
9–10	197 (43.0%)	62 (53.9%)	51 (44.7%)	42 (36.8%)	42 (36.5%)	
Baseline NIHSS	16.0 ± 4.1	15.9 ± 4.0	15.0 ± 3.9	15.5 ± 3.7	17.6 ± 4.4	<0.001
**Procedural variables**						
Bridging treatment	197 (43.0%)	52 (45.2%)	47 (41.2%)	56 (49.1%)	42 (36.5%)	0.252
rescue treatment	75 (16.4%)	22 (19.1%)	19 (16.7%)	19 (16.7%)	15 (13.0%)	0.663
PTR time, minute	47.0 (30.0–70.0)	44.0 (28.0–69.0)	51.5 (33.2–71.5)	45.0 (31.0–65.0)	45.0 (27.0–70.5)	0.487
OTR time, minute	270.1 ± 83.5	266.6 ± 88.5	283.5 ± 87.8	264.0 ± 76.5	266.3 ± 80.3	0.264
Continuous intravenous antihypertensive agents	353 (77.1%)	77 (67.0%)	81 (71.1%)	93 (81.6%)	102 (88.7%)	<0.001
**Blood pressure variables**						
MAP mean	88.6 ± 7.9	90.6 ± 9.4	92.4 ± 9.2	91.3 ± 8.6	88.6 ± 7.9	0.011
**Outcomes**						
90 day-mRS,						<0.001
0–2	251 (54.8%)	75 (65.2%)	71 (62.3%)	62 (54.4%)	43 (37.4%)	
3–6	207 (45.2%)	40 (34.8%)	43 (37.7%)	52 (45.6%)	72 (62.6%)	
30 day-mortality						<0.001
alive	397 (86.7%)	109 (94.8%)	103 (90.4%)	102 (89.5%)	83 (72.2%)	
dead	61 (13.3%)	6 (5.2%)	11 (9.6%)	12 (10.5%)	32 (27.8%)	
sICH						0.101
no	438 (95.6%)	113 (98.3%)	111 (97.4%)	108 (94.7%)	106 (92.2%)	
yes	20 (4.4%)	2 (1.7%)	3 (2.6%)	6 (5.3%)	9 (7.8%)	

Data reported as mean ± SD or median (IQR) for continuous variables or n (%) for categorical variables. MAP, mean arterial pressure; SD, Standard Deviation; IQR, Interquartile Range; ASPECTS, Alberta Stroke Program Early CT Score; NIHSS, National Institutes of Health Stroke Scale; ICA, Internal Carotid Artery; MCA, Middle Cerebral Artery; PTR, Puncture To Recanalization; OTR, Onset to Recanalization.

Participants with higher BPV tended to be older, men, had lower ASPECT scores, higher baseline NIHSS scores, hypertension, and showed higher usage of continuous intravenous antihypertensive agents.

### Outcomes

**Figure 2:**
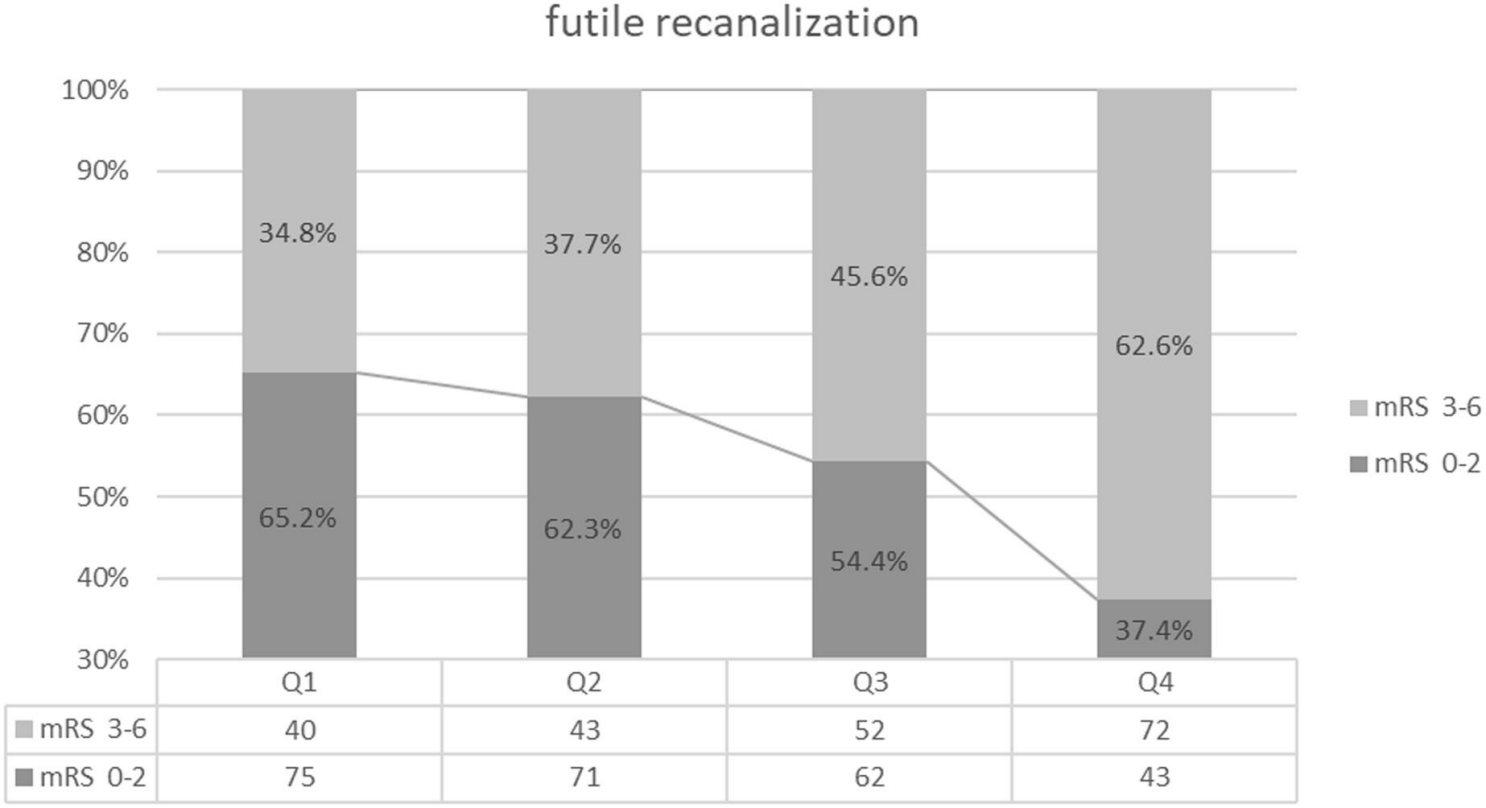
Futile recanalization comparison among the quartile groups.

**Figure 3:**
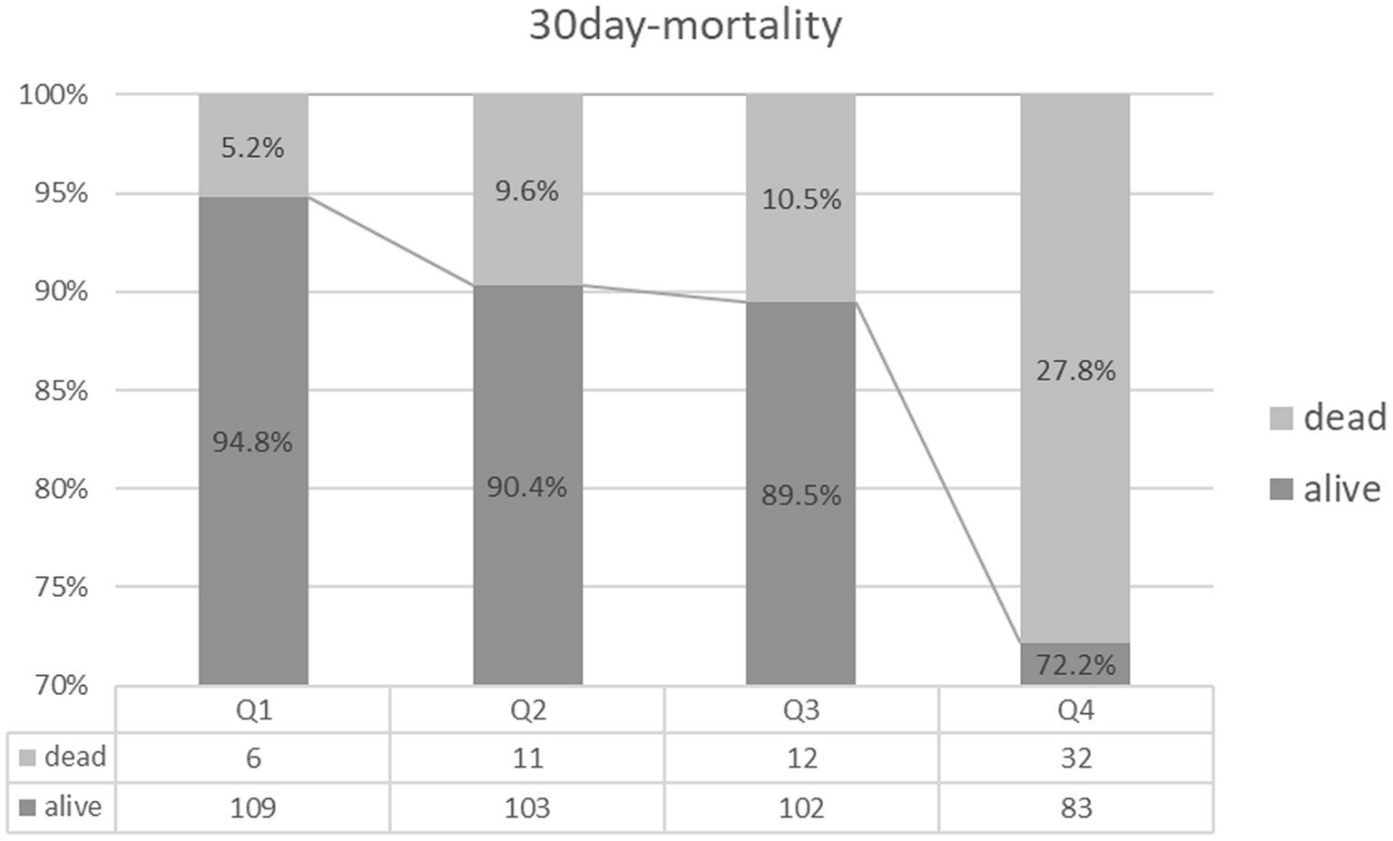
30 day-mortality comparison among the quartile groups.

**Figure 4:**
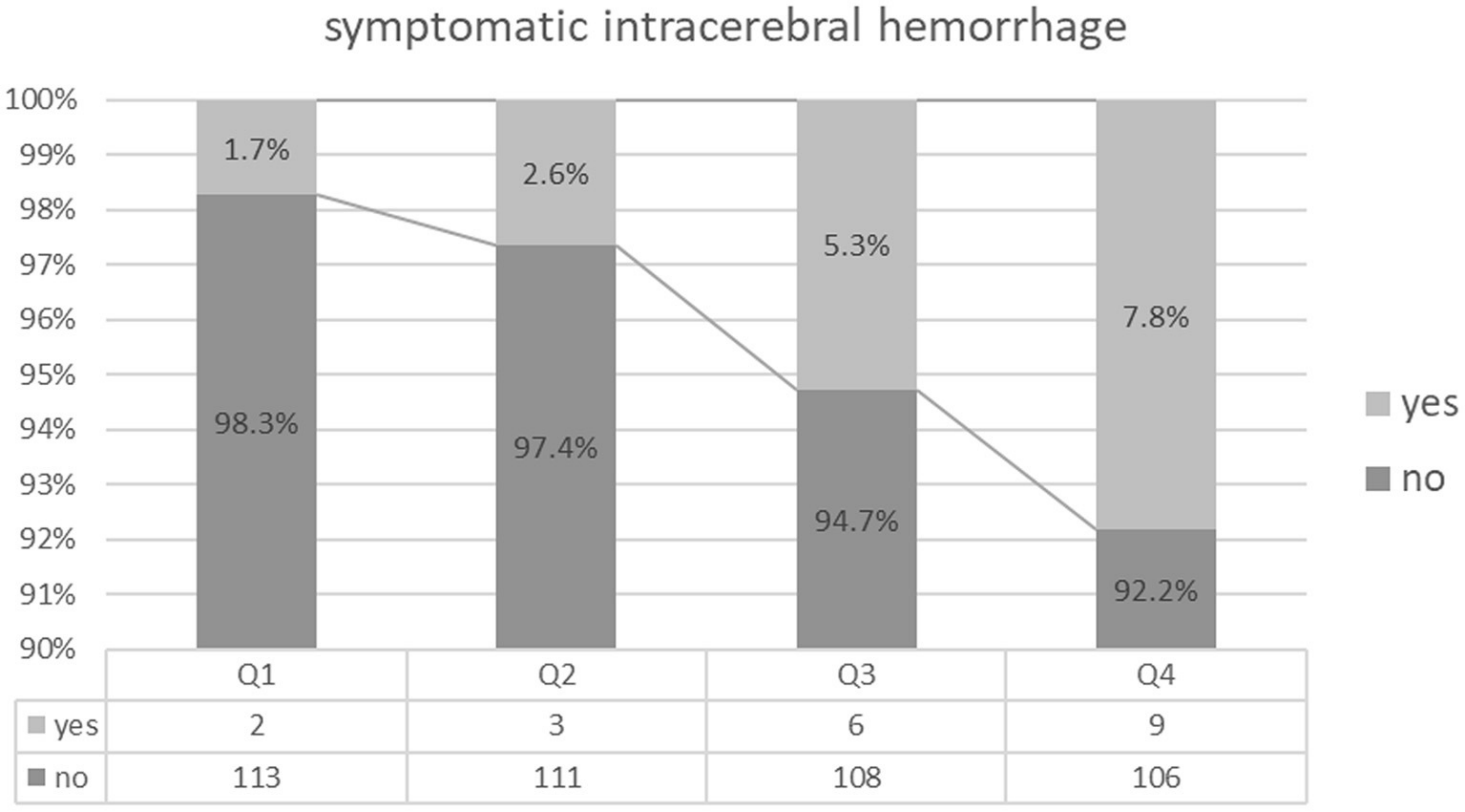
Symptomatic intracerebral hemorrhage comparison among the quartile groups.

A total of 207 (45.2%) patients had unfavorable functional outcomes (mRS score 3–6) at 90 days; 61 (13.3%) patients died within 30 days, and 20 (4.4%) patients experienced sICH (Table 1). Patients with higher BPV tended to have a higher prevalence of futile recanalization (Q1: 34.8%; Q2: 37.7%; Q3: 45.6%; Q4: 62.6%; *P* < 0.001), mortality (Q1: 5.2%; Q2: 9.6%; Q3: 10.5%; Q4: 27.8%; *P* < 0.001), and sICH (Q1: 1.7%; Q2:2.6%; Q3: 5.3%; Q4: 7.8%; *P* = 0.101) (Table 1 and Figures 2–4).

The results of the univariate analyses are summarized in Supplementary Table S1. Univariate analyses revealed an increased incidence for each outcome in patients with higher BPV (*P* < 0.05). Each 1 mmHg increment of MAP SD was associated with the following increases: 17% in futile recanalization (95% confidence interval [CI: 1.09 to 1.26); 35% in 30 day-mortality (95% CI: 1.22 to 1.48); and 23% in symptomatic intracerebral hemorrhage (95% CI: 1.10 to 1.37). No significant correlation was found between higher MAP means and each outcome (*P* > 0.05).

Smooth curve fitting plots (Supplementary Figures S1–S3) suggest that age, NIHSS score, PTR time, and OTR time were linearly related to all outcomes. While, MAP mean was linearly related to futile recanalization and symptomatic intracerebral hemorrhage instead of 30-day mortality. Thus, nonlinear regression (curve fit) was used for MAP mean in the multivariable analysis between 30-day mortality.

According to the above results, we performed a multivariate logistic analysis to further explore MAP SD as a prognostic marker. In the multivariable analysis, and the data with and without adjustment are listed in Table 2. The MAP SD level was an independent risk factor for poor functional outcome at 90 days in Model I (OR adj = 1.37, per 1-SD increase, 95% CI: 1.08–1.72, *P* = 0.009) and Model II (OR adj = 1.31, per 1-SD increase, 95% CI: 1.01–1.68, *P* = 0.039). Multivariable logistic regression also revealed a significant association between MAP SD level and 30-day mortality (OR adj = 1.80, per 1-SD increase, 95% CI: 1.29–2.50, *P* = 0.001, Model II) and sICH (OR adj = 1.69, per 1-SD increase, 95% CI: 1.15–2.47, *P* = 0.007, Model II).

**Table 2:** Multivariable regression of MAP SD associated with clinical outcomes.

**Outcomes**	**Crude model**		**Model I**		**Model II**	
	**OR (95% CI)**	***P* Value**	**OR (95% CI)**	***P* Value**	**OR (95% CI)**	***P* Value**
Futile recanalization						
MAP SD (mmHg)	1.17 (1.09, 1.26)	<0.001	1.11 (1.03, 1.21)	0.009	1.10 (1.00, 1.20)	0.039
MAP SD (per 1 SD)	1.59 (1.29, 1.95)	<0.001	1.37 (1.08, 1.73)	0.009	1.31 (1.01, 1.68)	0.039
30-day mortality						
MAP SD (mmHg)	1.35 (1.22, 1.48)	<0.001	1.26 (1.13, 1.40)	<0.001	1.22 (1.09, 1.37)	0.001
MAP SD (per 1 SD)	2.36 (1.78, 3.14)	<0.001	1.95 (1.43, 2.65)	<0.001	1.80 (1.29, 2.50)	0.001
Symptomatic intracerebral hemorrhage						
MAP SD (mmHg)	1.23 (1.10, 1.37)	<0.001	1.20 (1.06, 1.36)	0.005	1.20 (1.05, 1.36)	0.007
MAP SD (per 1 SD)	1.81 (1.31, 2.52)	<0.001	1.70 (1.18, 2.45)	0.005	1.69 (1.15, 2.47)	0.007

Crude model adjust for: None.

Model I adjust for: Male, Age, Hypertension, Diabetes mellitus, Atrial fibrillation, Level of occlusion on DSA imaging, ASPECTS, Baseline NIHSS.

Model II adjust for: Male, Age, Hypertension, Diabetes mellitus, Atrial fibrillation, Level of occlusion on DSA imaging, ASPECTS, Baseline NIHSS, Puncture to recanalization, Onset to recanalization, rescue treatment, Continuous intravenous antihypertensive agents, Bridging treatment, MAP mean (Smooth only for 30-day mortality).

**Figure 5:**
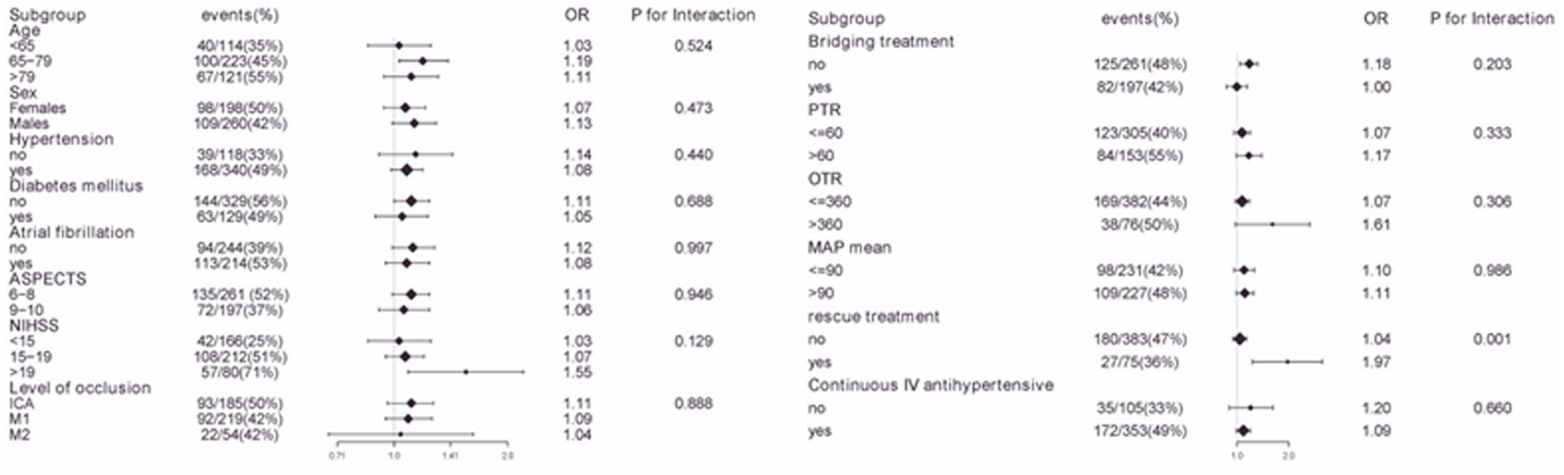
Interaction test for the effect of MAP SD on futile recanalization in different subgroups after adjusting for confounding factors. The above model adjusted for confounding factors in Table 2 (Model II). In each case, the model is not adjusted for the stratification variable.

**Figure 6:**
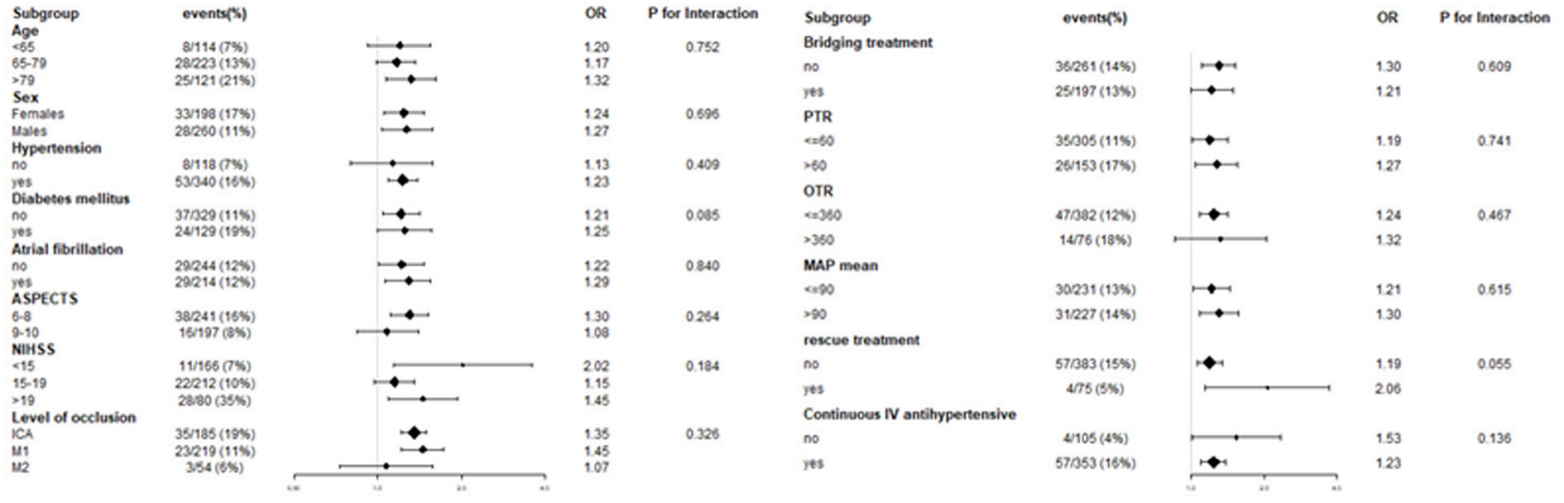
Interaction test for the effect of MAP SD on 30-day mortality in different subgroups after adjusting for confounding factors. The above model adjusted for confounding factors in Table 2 (Model II). In each case, the model is not adjusted for the stratification variable.

To evaluate other potentially influencing factors, we conducted a sub-analysis by stratifying patients according to age, sex, hypertension, diabetes mellitus, atrial fibrillation, and level of occlusion; ASPECT score, baseline NIHSS score, PTR time, OTR time, rescue treatment, continuous intravenous antihypertensive agents, bridging therapy, and mean MAP, as presented in Figures 5, 6. The number of patients with sICH was low; therefore, subgroup analysis was not performed. Notably, all subgroups demonstrated a similar relationship (all OR>1) between MAP SD level and post-operative futile recanalization and 30 day-mortality.

**Figure 7:**
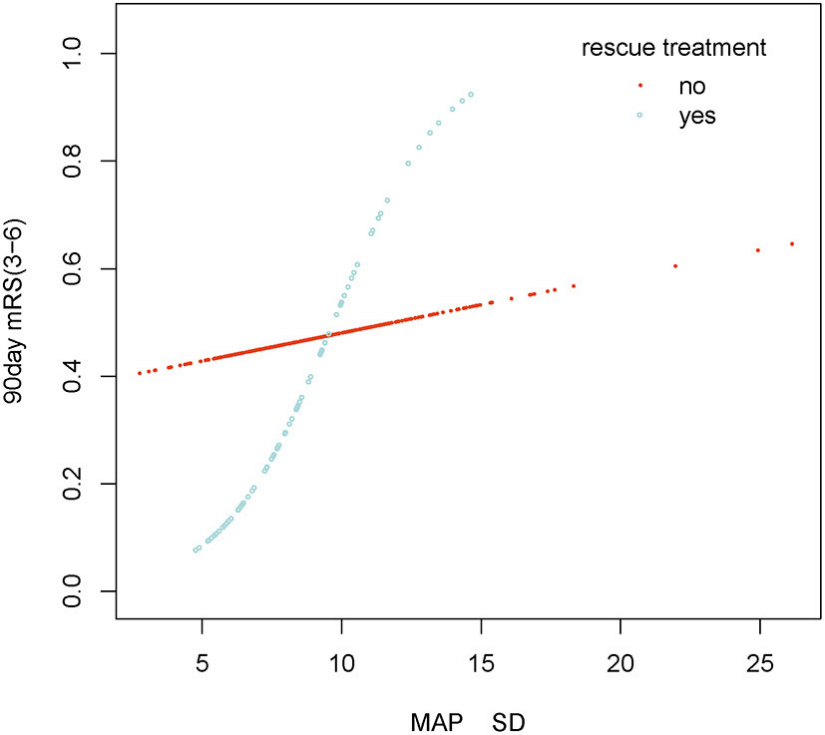
Smooth curve fitting of MAP SD and futile recanalization in rescue treatment subgroup. Adjustment variables: Male, Age, Hypertension, Diabetes mellitus, Atrial fibrillation, Level of occlusion on DSA imaging, ASPECTS, Baseline NIHSS, Puncture to recanalization, Onset to recanalization, rescue treatment, Continuous intravenous antihypertensive agents, Bridging treatment, MAP mean.

The stratified analyses of futile recanalization between each subgroup are shown in Figure 5. The interaction analysis revealed that rescue treatment played an interactive role in the association between BPV and futile recanalization (Figure 7). The patients who underwent rescue treatment had a higher odds ratio (OR) between BPV and futile recanalization (OR = 1.97; 95% CI, 1.23–3.14; *P* = 0.001) than those without rescue treatment (OR = 1.04; 95% CI, 0.95–1.14). The stratified analyses of 30-day mortality between each subgroup are shown in Figure 6. Logistic regression analysis did not demonstrate a significant relationship for 30 day-mortality between BPV and all subgroups (*P* > 0.05).

## Discussion

Our study showed that the successful recanalization (TICI 2b-3) rate of patients with LVO treated with MT was above 94%. The lower futile recanalization rate in our study compared to that in the HERMES study (2) (45.2 vs. 54.0%, respectively) may be attributed to the different periods in which the two studies were conducted (2017 vs. 2014), considering the advances in surgical devices and change in choice of surgical procedures [enhanced rescue treatment may improve neurological outcomes (23)]. Our study demonstrated similar 30-day mortality (13.3 vs. 15.3%, respectively) and sICH (4.4 vs. 4.4%, respectively) to the HERMES study. In this study, short-term BPV was significantly associated with adverse clinical outcomes.

BP is one of the strongest clinical decision tools (24, 25). The ASA guidelines recommended a BP target of 180/105 mmHg after MT as a reasonable extrapolation from intravenous thrombolysis literature (6). However, recanalization rates with MT are much higher than those of intravenous thrombolysis alone, and it remains unclear whether BP targets for patients who undergo MT should be the same. Given the considerable interindividual differences in hypertension history and collateral circulation, an individualized optimal BP may be more suitable than an absolute BP level (26).

Mechanisms such as exhausted collateral circulation, steal effects, and impaired cerebral autoregulation are more pronounced in patients with LVO stroke. Within a certain range, an almost linear relationship exists between cerebral blood flow and BP. The effects of inapt BP are more likely to be felt to a greater extent in the brain, which can lead to detrimental consequences (27). The 24-h postoperative MAP in this study was 89.9 mmHg, which is similar to the MAP target recommended by previous animal studies, clinical studies, and meta-analyses (28–30). However, the optimal mean MAP was not associated with favorable outcomes in our study.

Increased BPV is detrimental to functional outcomes after ischemic stroke (10); however, 24-h short-term BPV (assessed using ambulatory BP monitoring) shows a trend toward favorable outcomes after MT (31). MAP, a combination of SBP and DBP, is a more reliable biomarker for perfusion pressure in the brain (32). Considering that SBP or DBP is vulnerable to short fluctuations, we chose MAP as a predictive marker instead.

After adjusting for critical prognostic covariates of stroke, our study found that BPV was overall associated with unfavorable functional outcomes, which is consistent with the results of two recent studies. Secondary analysis of the BEST Study (a prospective, multicenter cohort study) indicated that patients with an increase in BPV are predisposed to poorer outcomes (90-day mRS 3–6) (13). Another prospective cohort study from Portugal showed that BPV was independently associated with poor functional outcomes at 90 days (19). However, our findings are inconsistent with a *post hoc* analysis of the BP TARGET trial, which showed that BPV was not associated with functional outcomes or sICH (19).

This discrepancy may be due to the following: (1) The number of BP measurements in the BP TARGET trial (15–16) was less than that in the other studies (24–37). The SBP SD/DBP SD (3.8–5.3/4.4–4.7 mmHg) in the BP TARGET trial were lower than those in the BEST study (13.9/10.4 mmHg), Portugal (13 /9 mmHg), and our study (12.5 /9.1 mmHg). Therefore, the data from the BP TARGET trial may fail to reflect actual BP fluctuations. (2) There were differences in the statistical methods (different adjusted covariates) and study populations among these studies. (3) The rate of internal carotid artery (ICA) occlusion in the BP TARGET trial (26%) was lower than that in our study (40%). Our subgroup analysis found that BPV after MT had a more substantial effect on patients with occlusion of the extracranial internal carotid artery when compared to occlusion of the intracranial middle cerebral artery. The differences in curvature and hemodynamic changes between the extracranial and intracranial arteries are the reason for this difference. Therefore, BP after recanalization has a more significant impact on the extracranial internal carotid artery (33). However, this explanation should be interpreted cautiously as our study was not a randomized trial to investigate the efficacy of BPV-lowering therapy.

Our study found that rescue treatment was an interaction factor between BPV and futile recanalization. Some published studies found that the BPV of patients with carotid artery stenting (CAS) before surgery was higher than that after surgery due to the compression of the stent on the carotid artery which affected the pressure. Therefore, it can be deduced that CAS could reduce BPV (34). A similar finding was made in our study, where the postoperative BPV and futile recanalization rates in the rescue treatment group (8.7 ± 2.4 vs. 9.1 ± 3.0 mmHg and 36 vs. 47%, respectively) were lower than that in the other group. Additionally, a Korean multicenter retrospective cohort study indicated that rescue stenting was independently associated with enhanced outcomes without an increase in sICH or mortality. This study found that patients in a rescue treatment group were younger, had lower NIHSS scores, more ICA occlusion, and had less arterial fibrillation than those in a non-rescue treatment group (35). In our study, the effect size remained unchanged after adjusting for confounders. Therefore, patients with higher BPV may have poorer outcomes, especially those who undergo rescue treatment.

Compared with previous studies, although our sample size was not significantly large, we used curve fitting and sensitive analysis. This lends credence to the association between BPV and functional outcomes and accuracy in identifying BPV vulnerable subgroups. We also demonstrated a linear association between BPV and each outcome.

Our study had several limitations. First, as this was a single-center study, our findings may not be generalizable to all settings. For the treatment of thrombectomy, SWIM (Solitaire stent retriever in combination with intracranial support catheter aspiration for MT) technology is the preferred choice at our center (36), while other centers may adopt conventional procedures, leading to differences in study results. Second, we measured BPV only after successful recanalization. BPV measurements before and during thrombectomy operations might be of great value for evaluating the association between BPV and outcomes. Third, our findings are based on a retrospective observational study. Additional randomized studies are warranted to confirm the causality of these observational results.

## Conclusion

Our results showed that increased BPV after MT was associated with postoperative adverse events. Identification of patients with higher BPV has the potential to inform clinical managements, including transfer to the NICU, closer observation, earlier imaging follow-up for the detection of sICH, and decompressive hemicraniectomy surgery. Stricter management of BPV is required for patients who undergo rescue treatment.

## Data availability statement

The original contributions presented in the study are included in the article/Supplementary material, further inquiries can be directed to the corresponding authors.

## Ethics statement

The studies involving human participants were reviewed and approved by Ethics Committee of Shanghai Tenth People's Hospital. The patients/participants provided their written informed consent to participate in this study.

## Author contributions

QZ and JH: conception, design, and administrative support. YL and RS: provision of study materials or patients. RS: collection and assembly of data. XZ and JH: adjudicated the radiological findings. WL: data analysis and interpretation. All authors: manuscript writing and final approval of manuscript.

## Funding

This study is supported by the Shanghai Municipal Key Clinical Specialty (No. shslczdzk06102).

## Conflict of interest

The authors declare that the research was conducted in the absence of any commercial or financial relationships that could be construed as a potential conflict of interest.

## Publisher's note

All claims expressed in this article are solely those of the authors and do not necessarily represent those of their affiliated organizations, or those of the publisher, the editors and the reviewers. Any product that may be evaluated in this article, or claim that may be made by its manufacturer, is not guaranteed or endorsed by the publisher.
