# Supplementary material for: Association between blood pressure variability and clinical outcomes after successful recanalization in patients with large vessel occlusion stroke after mechanical thrombectomy
Source: Front Neurol. 2022 Aug 10;13:967395. doi: 10.3389/fneur.2022.967395 (PMC9399916; doi:10.3389/fneur.2022.967395)
Supplement: Supplementary file 1 [file Data_Sheet_1.docx]

Supplementary Materials


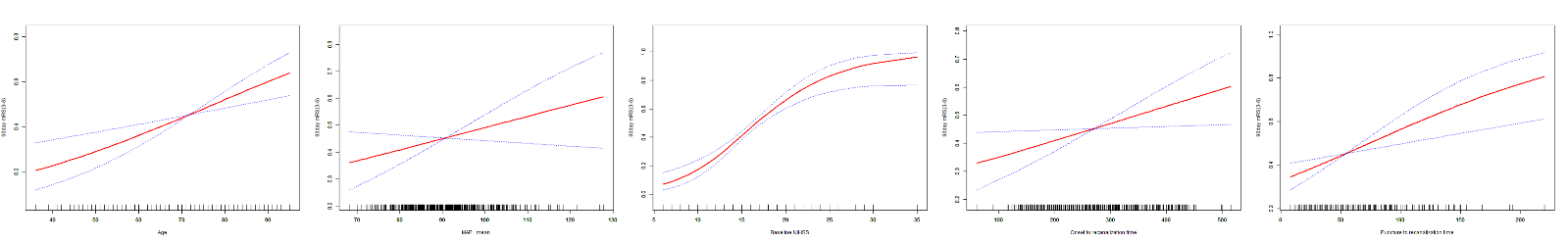


Figure S1 Smooth curve fitting of continuous variables (age, MAP mean, PTR, OTR, Base NIHSS) and futile recanalization


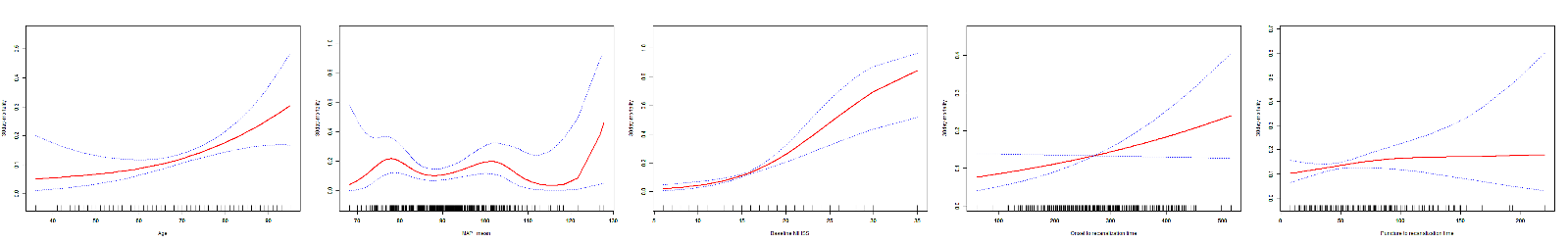


Figure S2 Smooth curve fitting of continuous variables (age, MAP mean, PTR, OTR, Base NIHSS) and 30day-mortality


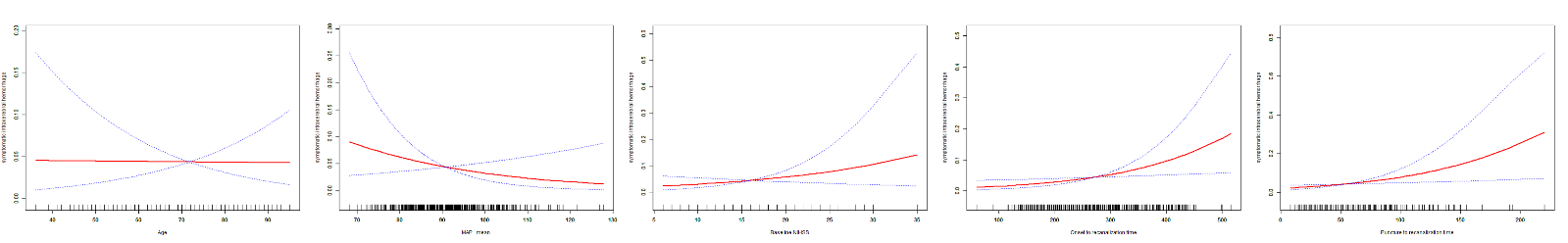


Figure S3 Smooth curve fitting of continuous variables (age, MAP mean, PTR, OTR, Base NIHSS) and symptomatic intracerebral hemorrhage

Solid red line represents the smooth curve fit between variables. Blue bands represent the 95% of confidence interval from the fit. Continuous variables were curve-fitted. Age, NIHSS score, PTR, and OTR were linearly related to all outcomes. MAP mean was linearly related to futile recanalization and symptomatic intracerebral hemorrhage instead of 30-day mortality.

Table S1 Univariate analysis for outcomes

|  | Statistics | 90-day mRS (3-6) | | 30-day mortality | | symptomatic intracerebral hemorrhage | |
| --- | --- | --- | --- | --- | --- | --- | --- |
|  |  | OR (95%CI) | P-value | OR (95%CI) | P-value | OR (95%CI) | P-value |
| MAP SD, mmHg | 9.1±2.9 | 1.17 (1.09, 1.26) | <0.001 | 1.35 (1.22, 1.48) | <0.001 | 1.23 (1.10, 1.37) | <0.001 |
| Age, year | 71.7±10.7 | 1.03 (1.01, 1.05) | <0.001 | 1.04 (1.01, 1.07) | 0.003 | 1.00 (0.96, 1.04) | 0.960 |
| Male, % |  |  |  |  |  |  |  |
| Females | 198 (43.2%) | Reference |  | Reference |  | Reference |  |
| Males | 260 (56.8%) | 0.74 (0.51, 1.07) | 0.107 | 0.60 (0.35, 1.04) | 0.068 | 0.49 (0.20, 1.23) | 0.129 |
| Hypertension, % |  |  |  |  |  |  |  |
| No | 118 (25.8%) | Reference |  | Reference |  | Reference |  |
| Yes | 340 (74.2%) | 1.98 (1.28, 3.07) | 0.002 | 2.54 (1.17, 5.51) | 0.019 | 2.02 (0.58, 7.01) | 0.269 |
| Diabetes mellitus, % |  |  |  |  |  |  |  |
| No | 329 (71.8%) | Reference |  | Reference |  | Reference |  |
| Yes | 129 (28.2%) | 1.23 (0.82, 1.84) | 0.327 | 1.80 (1.03, 3.16) | 0.039 | 1.10 (0.41, 2.92) | 0.852 |
| Atrial fibrillation, % |  |  |  |  |  |  |  |
| No | 244 (53.3%) | Reference |  | Reference |  | Reference |  |
| Yes | 214 (46.7%) | 1.79 (1.23, 2.59) | 0.002 | 1.30 (0.76, 2.24) | 0.336 | 0.93 (0.38, 2.29) | 0.874 |
| Level of occlusion, % |  |  |  |  |  |  |  |
| ICA | 185 (40.4%) | Reference |  | Reference |  | Reference |  |
| MCA 1 | 219 (47.8%) | 0.72 (0.48, 1.06) | 0.097 | 0.50 (0.29, 0.89) | 0.018 | 0.55 (0.22, 1.37) | 0.197 |
| MCA 2 | 54 (11.8%) | 0.68 (0.37, 1.26) | 0.219 | 0.25 (0.07, 0.85) | 0.027 | 0.00 (0.00, Inf) | 0.991 |
| ASPECTS, % |  |  |  |  |  |  |  |
| 6-8 | 261 (57.0%) | Reference |  | Reference |  | Reference |  |
| 9-10 | 197 (43.0%) | 0.54 (0.37, 0.78) | 0.001 | 0.42 (0.23, 0.78) | 0.005 | 0.70 (0.28, 1.80) | 0.461 |
| Baseline NIHSS | 16.0±4.1 | 1.24 (1.17, 1.31) | <0.001 | 1.22 (1.14, 1.31) | <0.001 | 1.07 (0.97, 1.18) | 0.195 |
| Bridging treatment, % |  |  |  |  |  |  |  |
| No | 261 (57.0%) | Reference |  | Reference |  | Reference |  |
| Yes | 197 (43.0%) | 0.78 (0.53, 1.13) | 0.182 | 0.91 (0.53, 1.57) | 0.731 | 0.70 (0.28, 1.80) | 0.461 |
| rescue treatment, % |  |  |  |  |  |  |  |
| No | 383 (83.6%) | Reference |  | Reference |  | Reference |  |
| Yes | 75 (16.4%) | 0.63 (0.38, 1.06) | 0.082 | 0.32 (0.11, 0.92) | 0.034 | 0.90 (0.26, 3.14) | 0.865 |
| PTR time, minute | 54.4±32.9 | 1.01 (1.00, 1.02) | 0.001 | 1.00 (1.00, 1.01) | 0.257 | 1.01 (1.00, 1.02) | 0.011 |
| OTR time. minute | 270.1±83.5 | 1.00 (1.00, 1.00) | 0.029 | 1.00 (1.00, 1.01) | 0.069 | 1.01 (1.00, 1.01) | 0.014 |
| Continuous intravenous antihypertensive agents, % |  |  |  |  |  |  |  |
| No | 105 (22.9%) | Reference |  | Reference |  | Reference |  |
| Yes | 353 (77.1%) | 1.90 (1.20, 3.00) | 0.006 | 4.86 (1.72, 13.73) | 0.003 | 1.72 (0.49, 5.99) | 0.394 |
| MAP mean, mmHg | 90.7±8.9 | 1.02 (1.00, 1.04) | 0.116 | 1.00 (0.97, 1.03) | 0.885 | 0.97 (0.91, 1.02) | 0.215 |

MAP, mean arterial pressure; SD, Standard Deviation; ICA, Internal carotid artery; MCA, middle cerebral artery; ASPECTS, Alberta Stroke Program Early CT Score; NIHSS, National Institutes of Health Stroke Scale; PTR, Puncture to recanalization; OTR, Onset to recanalization.
